# Academic Achievement: Influences of University Students’ Self-Management and Perceived Self-Efficacy

**DOI:** 10.3390/jintelligence10030055

**Published:** 2022-08-08

**Authors:** Mohammed Hasan Ali Al-Abyadh, Hani Abdel Hafeez Abdel Azeem

**Affiliations:** 1 Mental Health Department, College of Education, Prince Sattam bin Abdulaziz University, Alkharj 16273, Saudi Arabia; 2 College of Education, Thamar University, Thamar 87246, Yemen; 3 Quality Unit at the Higher Institute of Administrative Sciences, Janaklis, Al Buhayrah 22732, Egypt

**Keywords:** self-management skills, self-efficacy, academic achievement, university students, Egypt, KSA

## Abstract

Successful students are more than just those who have more effective and efficient learning techniques for acquiring and applying information. They can also motivate, evaluate, and adjust their behavior if they are not learning properly. Thus, the objective of this study was to investigate the influence of university students’ self-management during their learning experience and their self-efficacy on their academic achievement. Additionally, the study investigated the differences between the Egyptian and Saudi students’ perceptions of self-management skills and self-efficacy in their academic achievement within the two countries. A total of 889 students from two different Arab countries took part in the study (Egypt and the Kingdom of Saudi Arabia). The sample was given an online questionnaire to evaluate their self-management abilities, perceived self-efficacy, and academic achievement. A quantitative approach using SmartPLS-SEM was deployed. The findings demonstrate that self-management and self-efficacy have positive influences on students’ academic achievement in both countries. Further, self-management skills have been proven to influence self-efficacy, which in turn highly influences academic achievement. Moreover, the findings of the Multi-Group Analysis (MGA) did not report significant differences between the Egyptian and Saudi students in terms of their perception of self-management, self-efficacy, and academic achievement.

## 1. Introduction

In an effort to build the nation’s workforce for future rapid growth, the university education stage plays a vital role. According to Dev (2016), students’ learning outcomes, particularly at the university level, are a barometer of education’s success or ineffectiveness and a key predictor of youths’ and the nation’s future. Therefore, higher education should focus on the student’s whole development in terms of social, economic, and political environments, and it should be more than merely obtaining a certificate (Harris 2001). Successful students are not only those who have more efficient and effective learning techniques for acquiring and applying their information. They can also encourage themselves and assess and adjust their behavior if they are not learning appropriately (Kadiyono and Hafiar 2017). In this regard, Dembo (2004) identified six elements that students should manage to be good learners. These include self-motivation, learning techniques, social and physical environments, and time management. These elements serve as the foundation for structuring and integrating the essential skills to fulfill the academic expectations of university students learning. This concentration allows for the integration of both skill and academic-performance techniques. In addition, much self-motivation and self-discipline is required to achieve academic excellence (Kadiyono and Hafiar 2017).

Recently, researchers, families, policymakers, and planners have focused on student academic achievement (Dev 2016). Institutions should train students’ academic and life skills to ensure they can function at an appropriate learning level, according to previous research on comprehensive student development (Wood and Olivier 2004). This has inspired various studies into more effective methods of increasing academic standards, and it has been discovered that proper self-management for students of higher education, among other criteria, improves learning and academic accomplishment (Sikhwari 2014). Individuals with effective management skills, according to Kadiyono and Hafiar (2017), know where to place goals; how to solve problems effectively, think optimistically when presented with academic problems, utilize resources, manage their surroundings to meet their objectives; and may reflect on the causes of failure and establish objectives for future growth. Self-management is described as the ability to work efficiently toward significant goals while being adaptable in the face of difficulties (Agolla and Ongori 2009). According to Stan (2021), self-management is a multidimensional umbrella concept that combines the personal qualities of the individual on which it can work through a behavioral transformation process. In this essence, Agolla and Ongori (2009) claimed that students with higher levels of self-reported behavioral self-management report better levels of self-reported academic success and adaptability to change.

In the same line, previous studies have found that willingness to attempt and tenacity are some of the characteristics of students with a good level of self-efficacy (Ahmad and Safaria 2013). Students who have a good sense of self-efficacy will be able to pay close attention to, organize, and elaborate on content successfully due to their cognitive abilities (Heslin and Klehe 2006). Such students work consistently; if they are unable to follow the course, they devise efficient ways to overcome obstacles to reaching their goals. Self-efficacy, or belief in one’s talents and capacities for performance and learning, is an important characteristic of university students’ success (Hill 2002). Students who believe they can learn or complete an activity are more likely to accomplish the implementation of academic self-efficacy, study harder, persevere longer when faced with problems, and succeed at a better level than students who question their ability (Schunk and Pajares 2002). According to Bandura (1997), self-efficacy beliefs determine task selection, effort, perseverance, resilience, and accomplishment.

In summary, students’ ideas about their skills and the outcomes of their efforts have an important impact on how they behave. As a result, it is not surprising that a large body of research indicates that student skills impact learning and achievement (Meral et al. 2012). However, Novo and Calixto (2009) asserted that researchers do not provide deep and experimentally proven insights into the structure that lies at the foundation of learning processes and shape their growth, but rather about the challenges of the learning process. For example, several research studies in the United States (Kuhfeld et al. 2020), the Netherlands (Meeter 2021), Belgium (Maldonado and de Witte 2022), and Germany (Meeter 2021) have examined the difficulties imposed by COVID-19 on academic success (Schult et al. 2022). The majority of these studies looked at student standardized test scores before and after the spring 2020 lockdown and showed slight but substantial drops. Academic achievement is frequently related to successful students’ particular talents and abilities. According to Díaz-Morales and Escribano (2015), academic achievement is the result of the complex interplay of the psychological, economic, and social factors that contribute to students’ optimal growth. One of the most important indicators of a student’s performance is their academic achievement; hence, research into the elements influencing academic achievement has long been highly regarded (Rivkin et al. 2005). However, there is still a scarcity of studies on academic accomplishment and what factors should be developed (Kadiyono and Hafiar 2017), which is surprising given that the goal of learning (education) is to assist each student in achieving their desired level of growth.

Within the context of the above-mentioned introductory framework, this study (1) investigates the influence of university students’ self-management during their learning experiences and their self-efficacy on their academic achievement in two different countries (Egypt and KSA), all of which appear to be key aspects of the learning process. Moreover, the study is considered pioneer research that (2) investigates the differences between the Egyptian and Saudi students’ perceptions of self-management skills and self-efficacy in their academic achievement. However, of the massive research studies that investigated each variable of the current framework with another, the current framework is considered novel due to studying the current three variables together within two different contexts in two different countries on different continents. This study also offers valuable advice to students on self-concept and soft skills, as well as acts as a roadmap for future research by potential researchers. In actuality, improving educational achievements necessitates the development of soft skills to promote human capacities, which is required to encourage the individual’s growth (Levasseur 2013). Therefore, the interest in studying the aspects (skills) involved in academic performance stems primarily from the phenomenon’s complexity, the long-term impacts of which aim for high employability chances and good professional adaption.

## 2. Literature Review and Hypotheses Development

It is common knowledge that developing personal qualities during university education impacts a student’s later career and personal life since they are easily transferable. Subsequently, identifying individuals’ distinctive academic factors that contribute to achievement is critical since it aids academic success in higher education and potential career possibilities (Sanchez-Ruiz et al. 2016). Academic success is influenced by a wide range of factors. The “Coleman Report”, a report on academic achievement from a large-scale study, was published in the 1960s (Cheng et al. 2019), and numerous applications were produced based on this study as a result, which is essential for academic achievement difficulties. The elements influencing academic accomplishment can be loosely characterized as follows: psychological perceptions, student skills, and environmental perspectives (Dijkstra and Peschar 2003). Moreover, some researchers think that four elements influence academic achievement: individual, family, educational institution, and the environment; the factors involved in individual factors can be further divided into cognitive functioning, learning mindset, motivation, and self-aspiration (Hammouri 2004). Learning outcomes have become a phenomenon that everyone is interested in, which is why researchers have been working hard to uncover aspects that promote high academic achievement (Aremu and Sokan 2003). As a result, we present a theoretical background on this triangular relationship among self-management abilities, self-efficacy, and academic-achievement motivation among university students in this section.

### 2.1. The Role of Student Self-Management in Increasing Student Self-Efficacy

In a wide sense, self-efficacy is described as a person’s belief in his/her abilities to plan and carry out the steps required to achieve specific objectives (Bandura 1997). Bandura (2001) observed that students’ conduct is frequently best predicted by their ideas about their skills. Bandura (1997) proposed that self-efficacy influenced how students felt, thought, and acted. Self-efficacy, according to self-efficacy theory, is one’s belief in their capacity to plan and carry out a certain course of conduct to find a solution or complete a task (Eccles and Wigfield 2002). Thus, a student’s self-efficacy refers to an individual’s belief in the ability to learn and perform behavior at a particular level. In addition, a high level of students’ self-efficacy promotes skill development, capacity building, and resilience by promoting task motivation and commitment, hard-working spirit, longer endurance, and resilience, especially when faced with difficulties (Vermeiren et al. 2022).

In their conceptualization, Sharma and Nasa (2014) claimed that students’ abilities provide a method for explaining and predicting one’s feelings, thoughts, and behaviors, as well as organizing and carrying out courses of conduct to achieve certain goals. In this regard, self-management is described as the act of personally directing the dispositions, behavior, and recognition of persons toward achieving goals or tasks (Amini and Noroozi 2018). Self-management is an important tool for all types of learning, including materials and academic courses, as well as other curriculum areas and abilities. It refers to the tactics, procedures, and methods that we use to successfully direct the actions and behaviors of students during their studies (Jasim 2020). Self-management teaches students how to regulate their emotions, create objectives, and arrange themselves so that they may be powerful self-motivators (Amini and Noroozi 2018). This concept has a significant meaning, in that self-management affects one’s level of ability and the amount of tenacity required to achieve a tough goal (Bandura 2001). Therefore, self-management assists students in becoming effective students. Self-management enables students to stick to their strategies for completing tasks while remaining focused in the classroom (Jasim 2020). As a result, the researchers present the hypothesis below.

**Hypothesis** **1** **(H1).** *Students with high self-management are more likely to achieve a higher academic self-efficacy.*

### 2.2. The Role of Student Self-Management in Increasing Student Academic Achievement

In a determinate sense, self-management encompasses, among other things, self-discipline, self-control, self-regulation, willpower, ego strength, and effortful control (Duckworth and Kern 2011). Along the same line, self-management, according to CASEL (2018), is defined as the capacity to control an individual’s emotions, ideas, gratification, and actions to motivate oneself and strive toward academic and personal objectives. On the other hand, the approaches used to describe student achievement vary with the concept’s complexity and breadth. It refers to a student’s acquisitions in a structured academic setting, as evidenced by the value placed on academic performance expressed in grades, standardized test results, or teachers’ recognitions in evaluations (Erhuvwu and Adeyemi 2019). Academic achievement, operationally, indicates the set of learned knowledge, the degree of growth of capacities, and skills in the academic setting (Jeynes 2008). Most studies in this field emphasize the relationships between student skills and academic achievement (Di Fabio and Palazzeschi 2009) and occupational status (Deary et al. 2007). Sanchez-Ruiz et al. (2016) developed another argument for comparing and generalizing the findings of studies on the influence of students’ ability on academic accomplishment that refers to personality characteristics as indicators of academic achievement. Robbins et al. (2004) suggest a composite social model that includes individual skills, social engagement, and academic-related abilities to explain the mechanism of academic achievement. According to previous research, students who utilize self-regulation tactics (such as self-regulated learning, time management, goal planning, and metacognition) perform better in class (Stan 2021). In this essence, Claro and Loeb (2019) refer to self-management as the capacity to control an individual’s thoughts, emotions, and behaviors in a variety of settings. According to Balica et al. (2016) and Deming (2015), self-management is a powerful indicator of academic success, decision-making abilities, and competence in behavior modification. As a result, the following hypothesis is developed.

**Hypothesis** **2** **(H2).** *Students with high self-management are more likely to secure a higher academic achievement.*

### 2.3. The Role of Self-Efficacy in Enhancing Student Academic Achievement

Academic achievement was originally regarded as the most essential consequence of the formal academic experience (Kell et al. 2013); although there is little dispute about the importance of such achievements in student experience and later life, they are no longer the most important outcome (Colmar et al. 2019; Martinez et al. 2019).

Students’ views on their capacity to master new abilities and activities, frequently in a particular academic topic, are referred to as self-efficacy (Nasiriyan et al. 2011). In other words, Gardner (1983) defines a self-efficacious student as someone who believes in their ability to plan and carry out the steps necessary to achieve certain goals. According to Bandura (1997), perceived self-efficacy indicates people’s beliefs in their ability to achieve specific goals. Kryshko et al. (2022) argued that investigating the effect of self-efficacy on motivational adjustments to academic performance may be useful empirically. Thus, researchers pay little attention to this type of belief in effectiveness and its role in academic performance. Self-efficacy is a key element of Bandura’s (2001) social-cognitive theory, which asserts that self-influence profoundly influences behavior. It increases grit when faced with problems, promotes purposeful behaviors, supports long-term vision and develops self-regulation and allows for self-correction when required within the context of social-cognitive theory. Previous research has identified cognitive skills and academic self-efficacy as well-established determinants of academic performance (Köseoğlu 2015). According to Abouserie (1995), failure or success may be associated with weak or strong self-efficacy, and these links might influence university students’ performance. In previous research studies, belief in self-efficacy in various domains, along with various indicators of motivation and academic achievement, has emerged as an important determinant of students’ effective use of self-regulation skills and strategies (Kim et al. 2021; Kryshko et al. 2022). Several studies have demonstrated that self-efficacy is a reliable predictor of motivation and academic performance that is unaffected by time, place, or community (Duckworth et al. 2007). It is the motivational aspect of self-efficacy that appears to generate academic achievement (Ashwin 2006). According to Miller and Brickman (2004), excellent educational success is related to improved confidence in one’s abilities, which encourages students to accept more responsibilities for the effective completion of assignments and projects. As a result, strong self-efficacy is widely acknowledged as an essential predictor of work-related achievements. More specifically, Honicke and Broadbent (2016) examined 59 self-efficacy studies conducted at universities and discovered a modest relationship between academic achievement and self-efficacy. In a similar vein, Schunk and Zimmerman (Meral et al. 2012) identified a connection between academic achievement and self-efficacy, indicating that students’ academic achievement increases when they are taught to have stronger self-efficacy beliefs. As a consequence, we formulate the a hypotheses below.

**Hypothesis** **3** **(H3).** *Students who have a high level of self-efficacy are more likely to achieve higher academic achievement.*

**Hypothesis** **3** **(H4).** *Perceived self-efficacy positively mediates the relationship between perceived self-management and students’ academic achievement.*

**Figure 1:**
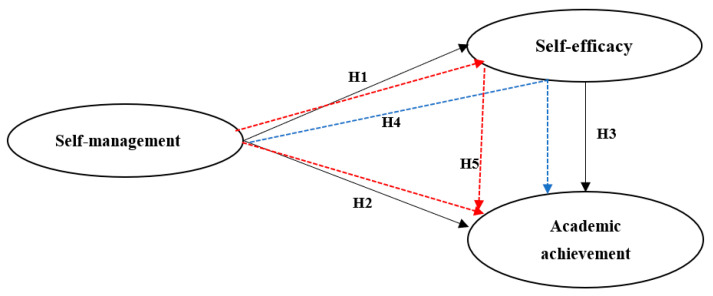
The research conceptual framework and hypotheses.

Trautwein et al. (2006) suggested an academic achievement model in which a variety of factors impact the completion of certain academic tasks. In addition to class and social characteristics, they looked at personal and intellectual qualities such as IQ, consciousness, knowledge, and attitude. In our study, we focused on prioritizing the role of personal and intellectual ability in terms of self-management and self-efficacy to better represent the factors facing college students (Figure 1). This adjustment is justified, since the involvement of student qualities (IQ, consciousness, knowledge) is expected to be equal at the same stage of education, especially if they are studying the same subject, even in different countries. This study was conducted on university students in two different countries (i.e., Egypt and the Kingdom of Saudi Arabia), to investigate the current research framework and to illustrate the differences between Egyptian and Saudi students, if applicable. Thus, we propose the following hypothesis.

**Hypothesis** **5** **(H5).** *There are no differences between Egyptian and Saudi students’ perceptions in terms of the direct and indirect relationships between self-management, self-efficacy, and academic achievement.*

## 3. Materials and Methods

### 3.1. Sampling and Data Collection

University students in Egypt and the Kingdom of Saudi Arabia (KSA) are the participants of the current study to align with the research objectives. Egyptian and Saudi universities were chosen for the field study due to the development of the education sector in both countries to achieve their visions for 2030. Additionally, the well-recognized economic development in all different sectors in both countries, (i.e., service and industrial) encourages students to build their academic careers to hunt for job opportunities after graduation. Finally, the authors of the current paper are faculty members in these countries. Thus, an online survey was established through Google docs targeting only 1600 students virtually representing Prince Sattam Bin Abdulaziz and King Saud University students in the KSA and University of Sadat City and Menoufia University in Egypt. We contacted the information technology unit of each university to disseminate the questionnaire to students after obtaining official approvals. The online survey link was sent to students via their academic emails. Of the 1600 respondents who received the online survey, we received 1005 surveys with a response rate of 62.8%, only 889 (KSA = 419; Egypt = 470) were eventually usable for the statistical analysis. About 116 surveys were excluded due to incomplete responses. Table 1 presents the demographics of the study’s participants.

### 3.2. Measurements

We deployed a quantitative approach to investigate the research hypotheses. Thus, the questionnaire was built based on a thorough revision of related research studies. Consequently, the questionnaire includes four categories: self-management, perceived self-efficacy, academic achievement, and respondents’ profiles. First, self-management was measured by 10 items adapted from (Öberg et al. 2019). Second, the perceived self-efficacy was measured by ten items retrieved from (Sukmak et al. 2001). Third, twenty items adapted from Turner (2007) were used to measure the motivations of students for academic achievement. Finally, the fourth section contains the students’ demographics. Additionally, all of the items in the questionnaire were assessed using five-point Likert scales ranging from “strongly disagree = 1” to “strongly agree = 5. The questionnaire was translated from English to the Arabic language to fit all students and to guarantee a full understanding of the questionnaire statements. To confirm the context validity of the questionnaire items before disseminating, the Arabic version of the questionnaire was retranslated into English. We conducted a pilot study on one hundred students in both countries to check the validity and reliability of the questionnaire. The findings of the refined draft of the questionnaire showed slight modifications to some Arabic words.

### 3.3. Data Analysis and Hypotheses Testing

The SmartPLS-SEM software, version 3.2.8 (Oststeinbek, Germany), was run to analyze the research data and test the hypotheses. The PLS technique has been extensively operationalized in all research disciplines for several reasons (Alsetoohy et al. 2019, 2021; Alsetoohy and Ayoun 2018). PLS is more suitable for small sample sizes, predictions, and the development of theories in research studies. Additionally, PLS is non-sensitive to the normality of data distribution. Finally, the PLS technique works well with models that have a large number of indicators. A two-step process (i.e., the measurement model and the structural model) was deployed to test the research hypotheses using Smart PLS-SEM software, version 3.2.8 (Oststeinbek, Germany) (Hair et al. 2012).

### 3.4. The Measurement Model

The validity and reliability of all latent variables of the study were assessed and checked to validate the research model relationships. To verify the internal reliability of the constructs, the Composite Reliability (CR) and Cronbach’s alpha were checked. The convergent validity of the model was assessed by the item loadings of the indicators, CR, and the average variance extracted (AVE). Furthermore, the Heterotrait–Monotrait (HTMT) ratio of correlation and the AVE were utilized to establish the discriminant validity. Finally, the variance inflation factor (VIF) was calculated to assess the collinearity of the constructions.

Table 2 illustrates that the Composite Reliability (CR) and Cronbach’s alpha values for all latent variables in the models were above the floor of .7 (Hair et al. 2012). Thus, the internal consistency of the research models was achieved. Additionally, the item loadings were above .60 (Hair et al. 2010). Two indicators (AA9 and AA10) were removed as their loadings were less than .60. The CR values were greater than .7 (Hair et al. 2012), and the AVE values were above the value of .5 (Fornell and Larcker 1981), which establishes the convergent validity. Likewise, the HTMT values ranged from .736 to .858, less than the floor of −.90 (Hair et al. 2012) (see Table 3). Therefore, discriminant validity was established for all models. Finally, the highest value of VIF is 4.331, which is lower than 5, confirming that there are no multicollinearity issues between the models’ constructs (Ringle et al. 2015).

### 3.5. Multigroup Analysis

After all the research models passed the robustness check using the measurement models’ assessment, we applied a non-parametric structural equation-modeling approach to analyze the differences between the Egyptian and Saudi students using Henseler’s MGA and the permutation test (Garson 2016; Henseler et al. 2016). Thus, the MICOM technique was run before the final step of the data analysis to test the invariance assessment to ensure the heterogeneity of the groups (Henseler et al. 2016). This technique was used to confirm that the same indicators were used for each measurement model and an acceptable reliability of each construct was obtained for both groups. Hence, two groups of students were created: Egyptians (*n* = 470) and Saudis (*n* = 419). Table 1 displays the assessment results of the measurement model between the two datasets of Egyptians (*n* = 470) and Saudis (*n* = 419) along with the total students’ model (*n* = 889). In step one, the assessment of configural invariance was achieved. Table 4 shows the results of the measurement invariance testing. The results of the compositional invariance assessment for Step two were established as none of the correlation (c) values are significantly different from 1. In Step 3, the composites’ equality of mean values and variances across the group was assessed. The results indicate that the confidence intervals of differences in mean values and variances partially include zero, which means the composite mean values and variances are partially equal. As such, achieving the establishment of the three steps of the MICOM procedure supports the partial measurement invariance of the two groups (Garson 2016; Henseler et al. 2016). This indicates that the pooled data for each group meets the requirement for comparing and interpreting any differences in structural relationships. Thus, further analysis for comparing and interpreting the MGA group-specific differences of PLS-SEM can be performed.

### 3.6. Testing the Research Hypotheses and Results

**Figure 2:**
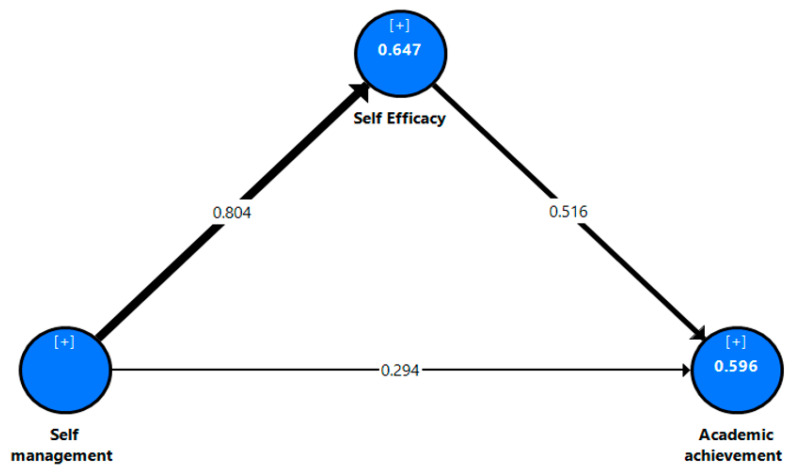
Results of the structural model with data from all students.

**Figure 3:**
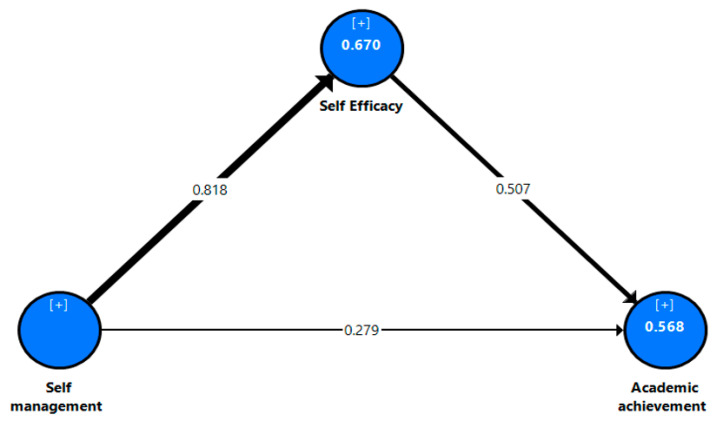
Results of the structural model with data from the Egyptian students.

**Figure 4:**
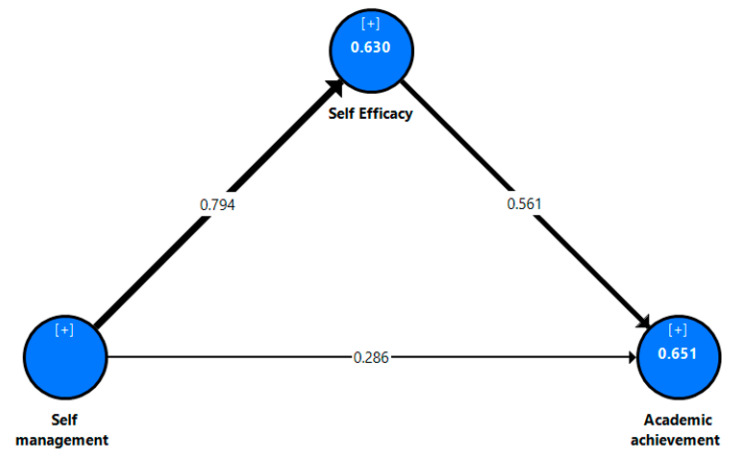
Results of the structural model with data from the Saudi students.

To assess the structural model of the current research study, we checked the R^2^ values, the p values, and the significance of the path coefficient (β) see Figure 2, Figure 3 and Figure 4. The results show that the R^2^ values achieved ranged between 56.8% to 67% for the dependent variable, which represents the substantial explanatory power of the current models (Chin 2010). The *p* values and the path coefficients refer to the statistical significances between the research variables. In general, the results of the research study show that perceived self-management has the strongest positive influence on the academic self-efficacy (β_all_ = .804, β_eg_ = .818, β_sa_ = .794; *p* = .000) of all students. This supports hypothesis 1 (H1). Moreover, the findings of the current study reveal that perceived self-management has positive effects on students’ academic achievement (β_all_ = .294, β_eg_ = .279, β_sa_ = .286; *p* = .000) in both countries. Thus, hypothesis 2 (H2) is supported. In the same context, the results of this study indicate that perceived self-efficacy is positively correlated with students’ academic achievement (β_all_ = .516, β_eg_ = .507, β_sa_ = .286; *p* = .000). Thus, hypothesis 3 (H3) is further supported.

To assess the significance/insignificance of the indirect effects of the current research model, bootstrapping tests with 5000 samples in SmartPLS-SEM were conducted to calculate the Bias-Corrected-Confidence Interval (BCCI), T-statistics, component weights, and observed significance values in the path coefficients to check the mediating effects of self-efficacy on the students’ academic achievement. The findings of the current study revealed a positive indirect significant relationship between perceived self-management (IV) and students’ academic achievement (DV) through perceived self-efficacy. Moreover, BBCI does not straddle zero between identified significant mediations, as shown in Table 5. The results report that perceived self-efficacy (β_all_ = .415, β_eg_ = .415, β_sa_ = .455; *p* = .000) positively mediates the relationship between self-management and students’ academic achievement, which supports hypothesis 4 (H4).

**Table 4:** Results of invariance measurement testing using permutation.

	Step 1	Step 2			Step 3						
	Configural Invariance	Original Correlation	5.0%	Compositional Invariance (Partial Measurement Invariance)	Mean Original Difference (Egypt–KSA)	Confidence Interval (2.5–97.5%)	Equality of Means	Variance Original Difference (Egypt–KSA)	Confidence Interval (2.5−97.5%)	Equality of Variance	Full Measurement Invariance
**Academic achievement**	**Established**	1.000	1.000	**Established**	−.033	(−.176, .180)	**Equal**	−.271	(−.298, .297)	**Equal**	**Established**
**Self-efficacy**	**Established**	1.000	1.000	**Established**	.221	(−.178, .185)	**Not Equal**	−.109	(−.266, .289)	**Equal**	**Established**
**Self-management**	**Established**	.999	.999	**Established**	.089	(−.176, .180)	**Equal**	−.245	(−.235, .247)	**Not Equal**	**Not Established**

As a prior step, the MGA was conducted using the Egyptian and Saudis datasets after completing the MICOM tests. In general, the MGA results showed non-significant differences between Egyptian and Saudis students for both direct relationships and indirect relationships of the research model, see Table 4. This supports hypothesis 5 (H5). Thus, the results of the total participant students in the current study (Egyptian and Saudi students) can be generalized.

## 4. Discussion

The current research sought to measure the relative impact of the self-management concept on modeling students’ academic achievement via self-efficacy.

On the one hand, for students of developed countries, there is a clear path from academic self-management, self-efficacy, student dedication, patience, and goal setting to ultimate academic performance (Bandura et al. 2001; Honicke and Broadbent 2016). Thus, the current research study examines the influence of self-management and self-efficacy on student academic achievement among students in two different developing countries. We attempted to overcome the shortcomings of previous studies in this area by (1) considering several theoretical and empirically distinct foundations of student achievement, (2) students’ self-management and self-efficacy, and (3) investigating predictors in two different domains, namely Egypt and the Kingdom of Saudi Arabia.

However, although the MGA results did not show significant differences between the Egyptian students (see Figure 2) and the Saudi students (see Figure 3), the results of Figure 1 (i.e., the total model) can be used to generalize this research results. The interpretation of the non-significant differences between the Saudi and Egyptian students may be due to both countries being in different regions and students speaking the same language (Arabic) and sharing the same traditions and customs. Additionally, a large number of Egyptian faculty members teach in Saudi universities, which in turn may lead to similar influences on students’ academic consciousnesses, knowledge, and academic accomplishments. These factors may contribute to diminishing the differences between students in both countries in terms of self-management, self-efficacy, and academic achievement. This finding is contrary to previous research studies (Oettingen 1997; Scholz et al. 2002), which confirmed that there was a cultural variation in how people felt about their abilities.

Among the predictor factors, students’ self-efficacy explained the most variance in academic achievement. It is considered that students’ self-efficacy assessments have a significant impact on their learning-process success. Students’ self-efficacy contributed significantly to the variation in the criteria in our study. It was revealed that students who are self-assured and more confident are more likely to achieve higher academic achievements, confirming that self-efficacy beliefs play an essential role in explaining academic achievement. The relative superiority of students’ self-efficacy in this investigation is consistent with the literature on the subject (e.g., Affuso et al. 2017; Honicke and Broadbent 2016; Köseoğlu 2015; Meral et al. 2012; Travis et al. 2020) and with several studies that have looked at the antecedents that influence academic accomplishment (e.g., Ashwin 2006; Hennig-Thurau et al. 2001). Crain (2005) claims that, when students have doubts about their abilities, they are less active and more likely to have no problems.

Students develop academic self-efficacy by evaluating and interpreting their task performance, which represents a self-judgment of competence (Bandura et al. 2001; Usher and Pajares 2009). Additionally, Ansong et al. (2019) argued that students’ self-efficacy is more likely to increase when students believe their academic abilities and efforts are successful and, conversely, are likely to diminish when they feel their efforts are insufficient. As a result, students with a high level of self-efficacy mastered their objectives, which included challenges and new information; performance quality, which included good grades; and outperforming peers. When they feel they are good at something, they work hard at it and stick with it despite failures (Crain 2005).

Moreover, self-management was also found to have a key impact on self-efficacy. According to our findings, the degree of self-efficacy determines a high percentage of the variation in the self-efficacy criteria, which is consistent with other studies (e.g., Di Fabio and Palazzeschi 2009; Stan 2021). Self-management is a broad concept that encompasses qualities such as self-efficacy. Self-management is widely recognized as one of the required abilities that drive students toward becoming more self-determined youths who can responsibly and proactively manage the elements of their lives, both in and out of educational contexts, according to King-Sears (2006). As a result, our study’s perspective is that students who can create objectives and employ various self-management tactics have better self-efficacy.

Furthermore, this study demonstrates that self-efficacy is a mediating factor in the relationship between self-management and academic achievement. Although analyses of the specialized literature confirm that self-management predicts student success (because the relationship with self-management is stronger than any other component of self-efficacy) (Stan 2021), our research results indicate that, without self-efficacy (mastery of skills and activities), academic achievement is relative. It might be claimed that academic self-efficacy is frequently used to prepare and carry out the procedures required to accomplish certain goals. Perceived self-efficacy, according to Bandura (1997), relates to students’ beliefs in their capacity to attain specified goals. So, the role of self-efficacy in explaining variation in academic achievement across students is a central theme in our study.

Furthermore, our research shows that students’ self-management has a modest influence on academic achievement. This outcome is consistent with the arguments of Kadiyono and Hafiar (2017), who believe that academic self-management may be utilized to motivate students to enhance their academic achievement, so that they can build a solid foundation to go forward and construct their futures. Nonetheless, given a well-established research background supporting self-management as an intervention, it appears that its usage among students must be encouraged by their instructors’ actions. Thus, when students are confident in their academic ability, they can set educational goals that drive them to academic excellence. On the other hand, students with little or no confidence in their abilities and capacities may be less likely to pursue higher levels of academic performance that require a higher level of effort, abilities, and skills; this confirms the findings of Ansong et al. (2019). In this regard, King-Sears (2006) argued that teachers play a critical role in enhancing students’ abilities to practice self-management.

## 5. Conclusions

The conclusions of this study have a variety of ramifications for educators, counselors, and students. This study attempted to investigate whether students’ self-management and self-efficacy produce excellent academic achievement when adopted by students working around a range of academic variables. The current study confirmed the significant relationships between self-management, self-efficacy, and academic achievement in two different domains (i.e., Egypt and KSA) through three models with identical significant results. Thus, academia and practitioners can use this research framework to guide their students to effective academic accomplishments. Additionally, our results did not show differences between students in terms of self-management, self-efficacy, and academic achievement according to country. This supports a fundamental conceptualization that students with different skills and motives can direct these positively toward their academic achievement regardless of their geographical domain and culture. Thus, the current study is considered a pioneer study that investigates the relationships between self-management, self-efficacy, and academic achievement among university students all in one model. This could be a guide for both students and educators who are seeking to optimize their (students’) academic achievements through self-management and efficacy. Additionally, this model was tested twice in two different countries which, in turn, helps generalize the results among all university students.

Due to the lack of orientation, self-management provides a fair to good degree of academic accomplishment, highlighting the need for treatments aimed at assisting students in developing a meaningful understanding of their self-management about their current views. The findings of this study confirm that self-management helps students control their impulses, set goals, organize themselves, and become strong self-motivators. Hence, students who can coordinate emotions and control and manage impulsivity stress are more likely to recognize goals and achieve them consistently. Additionally, students need to be aware of the purpose, the breadth, and the depth of self-management research and how expanding this skill can alleviate current problems. As a result, the current study elicits the role of educators, mentors, and counselors to empower and direct students’ motives, skills, and abilities to achieve both academic and life goals through facing and overcoming daily problems. Moreover, these findings affirmed that self-management is a powerful indicator of academic success, decision-making abilities, and competence in the behavior modification among students. This helps educators and students to modify students’ behaviors in a positive manner to establish academic achievement in both the short and long term. Nonetheless, the foundation of self-management plays a significant part in attaining students’ self-efficacy, due to its critical function in organizing all sorts of learning, including materials and academic courses. Such a finding is very noticeable in the overall evaluation of university students’ achievements. The results reveal that self-efficacy is a positive predictor of students’ academic achievement. Self-efficacy and academic achievement are reciprocally associated and mutually reinforcing, according to the mutual-effects model used in this study. Educators and university educators must create and use treatments that target self-management, self-efficacy, and academic achievement to put the model into effect. Finally, the positive relationship between the triangle-connection modeling could be used as a base for policymakers when establishing new curricula targeting efficient outcomes for students, educators, and the community.

Some limitations must be considered when evaluating the current study’s conclusions. Two distinct students’ behaviors were evaluated in this study, with different instructors adopting different teaching strategies. Future studies should aim to evaluate the triangle-connection modeling individually to obtain benchmark findings in each situation. The current study does not allow for a thorough conclusion about the underlying causes of the reciprocal impact of self-management, self-efficacy, and academic achievement. Further research should put to the test theoretically relevant antecedent models that might explain the relationships between self-management, self-efficacy, and academic achievement in greater depth. For example, engagement in supportive institutional–student connections in terms of teaching staff, teaching style, etc., can impact self-management, self-efficacy, and academic achievement all at the same time.

## Figures and Tables

**Table 1:** Sociodemographic characteristics of the students.

Characteristics	Frequency	%
Gender		
Male	446	50.2
Female	443	49.8
Nationality		
Saudi	419	47.1
Egyptian	470	55.9
Age		
18 years old	32	3.6
19 years old	100	11.3
20 years old	122	13.7
21 years old	253	28.5
22 years old	226	25.4
23 years old	114	12.8
24 years old	32	3.6
25 years old	10	1.1
26 years old	3	.03
27 years old	1	.01
Level		
Level 1	102	10.6
Level 2	114	14.7
Level 3	96	9.8
Level 4	126	12.9
Level 5	148	15.1
Level 6	148	15.1
Level 7	106	21.7
Accommodation		
Countryside	424	47.7
Urban	464	52.2
Missing	1	.01

**Table 2:** Assessment results of the measurement model.

Construct/Item	Item Loadings			Cronbach’s Alpha			CR			AVE		
	All	Egyptians	Saudis	All	Egyptians	Saudis	All	Egyptians	Saudis	All	Egyptians	Saudis
**Self-management**				**.907**	**.895**	**.920**	**.923**	**.914**	**.933**	**.546**	**.517**	**.583**
SM1: I have enough knowledge about my condition	**.655**	**.659**	**.664**									
SM2: I have good social support, which makes it easier for me	**.649**	**.601**	**.705**									
SM3: I have those who support me to make self-management.	**.694**	**.620**	**.768**									
SM4: I find joy in everyday life despite my stress	**.758**	**.711**	**.805**									
SM5: I know how to handle the stress in daily life	**.795**	**.785**	**.809**									
SM6: I have found good daily life	**.750**	**.712**	**.805**									
SM7: I have received a sufficient amount of information	**.767**	**.784**	**.750**									
SM8: I feel satisfied with my study.	**.728**	**.701**	**.760**									
SM9: I have a plan for how to deal with my illness	**.791**	**.774**	**.809**									
SM10: I have concrete plans for my future self-management	**.783**	**.821**	**.744**									
**Self-efficacy**				**.952**	**.952**	**.950**	**.958**	**.959**	**.957**	**.697**	**.702**	**.691**
SE1: I can always manage to solve different problems if I try hard enough	**.848**	**.878**	**.812**									
SE2: If someone opposes me, I can find the ways and means to get what I want.	**.818**	**.819**	**.813**									
SE3: It is easy for me to stick to my aims and accomplish my goals.	**.798**	**.784**	**.808**									
SE4: I am confident that I could deal efficiently with unexpected events.	**.847**	**.845**	**.850**									
SE5: Thank you for my resourcefulness how to handle unforeseen situations	**.821**	**.818**	**.824**									
SE6: I can solve most problems if I invest the necessary effort.	**.869**	**.880**	**.856**									
SE7: I can remain calm when facing difficulties because I can rely on my coping abilities	**.772**	**.736**	**.822**									
SE8: When I am confronted with a problem, I can usually find several solutions.	**.852**	**.851**	**.853**									
SE9: If I am in trouble, I can usually think of a solution.	**.870**	**.883**	**.853**									
SE10: I can usually handle whatever comes my way	**.849**	**.873**	**.819**									
**Academic achievement**				**.955**	**.950**	**.961**	**.960**	**.956**	**.965**	**.573**	**.548**	**.607**
**AA1:** I try to understand the course material rather than simply memorize it.	**.608**	**.641**	**.599**									
**AA2:** I want to make my family happy by succeeding in school	**.749**	**.727**	**.788**									
**AA3:** Getting good grades are important to me.	**.750**	**.690**	**.810**									
**AA4:** I am interested and pay attention during lectures.	**.767**	**.721**	**.817**									
**AA5:** Doing well in school is one of my main goals.	**.828**	**.819**	**.843**									
**AA6:** I am capable of getting a GPA of 3.5 or better.	**.766**	**.754**	**.789**									
**AA7:** I am persistent in the pursuit of my academic goals.	**.769**	**.761**	**.782**									
**AA8:** My grades are a higher priority than my social life is.	**.788**	**.771**	**.807**									
***AA9:*** *I participate in extra-curricular activities at the university.*	**------**	**------**	**------**									
***AA10:*** *I enjoy writing essays in which I can counter-argue a point.*	**-----**	**------**	**------**									
**AA11:** I complete my assignments well in advance	**.755**	**.738**	**.780**									
**AA12:** I take the time I need to prepare for exams	**.703**	**.693**	**.710**									
**AA13:** I would like to be seen as someone successful in school.	**.874**	**.887**	**.864**									
**AA14:** I want to show everyone what I can accomplish in school	**.704**	**.664**	**.743**									
**AA15:** I enjoy getting my marks back after an assignment or test	**.778**	**.756**	**.802**									
**AA16:** I enjoy writing tests	**.656**	**.643**	**.673**									
**AA17:** Others might consider me to be a “keener” in school.	**.642**	**.600**	**.704**									
**AA18:** I completed all the assignments, even the optional ones	**.754**	**.737**	**.770**									
**AA19:** I feel driven to achieve success in university.	**.875**	**.881**	**.876**									
**AA20:** I tend to be a perfectionist when it comes to my assignments.	**.805**	**.795**	**.814**									

NB. AA9 and AA10 in ***italic*** were dropped.

**Table 3:** Heterotrait–Monotrait Ratio (HTMT).

	ALL Students (*n* = 889)			Egyptians (*n* = 470)			Saudis (*n* = 419)		
	1	2	3	1	2	3	1	2	3
**1. Academic achievement**									
**2. Self-efficacy**	**.784**			**.761**			**.822**		
**3. Self-management**	**.754**	**.858**		**.736**	**.876**		**.773**	**.843**	

**Table 5:** Results of hypotheses.

Constructs	Path Coefficients (β)			Confidence Intervals Corrected Bias (2.5–97.5%)			MGA	Results	
	All	Egyptians	Saudis	All	Egyptians	Saudis	β_differ_	Full Model	MGA Model
**Self-management -> Self-efficacy**	.804 ***	.818 ***	.794 ***	(.759, .846)	(.750, .862)	(.721, .859)	.025	Yes	No
**Self-management -> Academic achievement**	.294 ***	.279 **	.286 ***	(.187, .408)	(.113, .423)	(.140, .455)	−.007	Yes	No
**Self-efficacy -> Academic achievement**	.516 ***	.507 ***	.561 ***	(.393, .626)	(.332, .668)	(.390, .708)	−.053	Yes	No
**Self-management -> Self-efficacy -> Academic achievement**	.415 ***	.415 ***	.445 ***	(.320, .508)	(.271, .552)	(.312, .566)	−.030	Yes	No

** *p* < 0.01; *** *p* < 0.001.

## Data Availability

Not applicable.
